# High-Flow Nasal Oxygen and Desaturation During Deep Sedation for Colonoscopy in Patients at Increased Respiratory Risk: A Propensity Score-Matched Cohort Study

**DOI:** 10.3390/jcm15218254

**Published:** 2026-10-23

**Authors:** M. Ángeles Alonso-Fernández, Óscar Martínez-González, Mónica García-Alonso, Carmen Martín-Parra, Susana Soto-Fernández, Blanca López-Matamala, Elena Marín-Alcolado, Madian Manso-Álvarez, M. José Pérez-Grueso, Miriam Chana-García, María de Lucas-Gallego, Rafael Blancas

**Affiliations:** 1 Critical Care Department, Hospital Universitario del Tajo, 28300 Aranjuez, Spain; mafernandez@salud.madrid.org (M.Á.A.-F.); mdelcarmen.martinpa@salud.madrid.org (C.M.-P.); mblanca.lopezm@salud.madrid.org (B.L.-M.); madian.manso@salud.madrid.org (M.M.-Á.); miriam.chana@salud.madrid.org (M.C.-G.); 2 Facultad de Ciencias Biomédicas y de la Salud, Universidad Alfonso X el Sabio, 28692 Villanueva de la Cañada, Spain; mgarciaalo.hcsc@salud.madrid.org (M.G.-A.); susana.sotofernandez@salud.madrid.org (S.S.-F.); mmarina@salud.madrid.org (E.M.-A.); mariajose.perezgrueso@salud.madrid.org (M.J.P.-G.); mlucasg@salud.madrid.org (M.d.L.-G.); 3 Fundación para la Investigación Biomédica e Innovación, Hospital Universitario Infanta Sofía, Hospital Universitario del Henares y Hospital Universitario del Tajo, 28702 San Sebastián de los Reyes, Spain; 4 Critical Care Department, Hospital General Universitario Gregorio Marañón, 28030 Madrid, Spain; oscar.martinezgonzal@salud.madrid.org; 5 Facultad de Medicina, Universidad Complutense de Madrid, 28040 Madrid, Spain; 6 Gastroenterology Department, Hospital Universitario del Tajo, 28300 Aranjuez, Spain

**Keywords:** high-flow nasal oxygen, procedural sedation, colonoscopy, obstructive sleep apnoea, propensity score matching, desaturation, oxygen delivery strategy

## Abstract

**Background/Objectives**: Hypoxaemia is the most common adverse event during procedural sedation. High-flow nasal oxygen (HFNO) may reduce desaturation through continuous positive airway pressure, anatomical dead space washout, and stable FiO_2_ delivery. This retrospective cohort study assessed the association between two oxygen delivery strategies—HFNO versus Venturi mask oxygen—and the incidence of desaturation (SpO_2_ <90% at any time) during deep sedation for colonoscopy in patients at increased respiratory risk. The two strategies differed simultaneously in flow rate, delivered FiO_2_ (0.50 vs. 0.40), device interface, and humidification; any observed difference cannot be attributed exclusively to the high-flow mechanism. **Methods**: A single-centre retrospective cohort study with propensity score matching (PSM) was performed, including 200 consecutive adult patients with established obstructive sleep apnoea (OSA) or high-risk STOP-BANG criteria undergoing deep sedation for colonoscopy, grouped by oxygen delivery strategy: HFNO via nasal cannula (50 L/min, FiO_2_ 0.50) versus Venturi mask (12 L/min, FiO_2_ 0.40). The estimand of interest was the average treatment effect on the treated (ATT). **Results**: After 1:1 nearest-neighbour PSM (calliper 0.2 SD of the logit propensity score), 71 matched pairs were obtained; 10 of 17 covariates achieved adequate balance (SMD < 0.10). In the matched cohort, desaturation occurred in 12.7% of HFNO patients versus 22.5% of controls (matched-pair OR 0.53, 95% CI 0.23–1.23; *p* = 0.211; McNemar test), an absolute risk difference of 9.8 percentage points (95% CI −1.5 to +21.1). The estimate was imprecise and the confidence interval included no association. A sensitivity analysis using conditional logistic regression yielded an adjusted OR of 0.52 (95% CI 0.22–1.21; *p* = 0.129). The sample provided only 30.8% statistical power; an estimated 474 total patients (237 per group, parallel group design) would be required for 80% power in a future confirmatory randomised trial. **Conclusions**: In this propensity-matched cohort, the observed desaturation rate was lower with HFNO than with Venturi mask oxygen, but the estimate was imprecise, the confidence interval included no association, and residual confounding precludes definitive conclusions. These findings are hypothesis-generating and should be evaluated in a prospectively designed, adequately powered randomised trial.

## 1. Introduction

Gastrointestinal endoscopy is one of the most frequently performed diagnostic and therapeutic procedures worldwide, with demand rising steadily as a result of colorectal cancer screening programmes targeting an increasingly elderly and comorbid population [1]. Deep sedation, most commonly propofol-based, has become the standard of care for endoscopic procedures, as it improves patient tolerance, facilitates procedural completion, and reduces discomfort [2,3,4]. Hypoxaemia is the most common sedation-related adverse event, arising primarily from respiratory centre depression, upper airway obstruction secondary to muscular hypotonia, and reduced chest wall compliance, all potentiated by increasing sedation depth [5,6,7]. Reported desaturation rates vary widely across studies, but events requiring airway intervention occur in approximately 6% of procedures in unselected populations, with higher rates in patients identified as high risk [8].

Established predictors of desaturation during sedated endoscopy include age over 60 years, ASA physical status ≥ III, body mass index (BMI) >30 kg/m^2^, pre-existing obstructive sleep apnoea (OSA), and significant cardiorespiratory comorbidities [9,10]. Patients with OSA and obesity are particularly vulnerable during deep sedation; sedative agents blunt microarousal reflexes that normally protect patients from pharyngeal collapse during sleep, and the combination of muscle relaxation and reduced respiratory drive further increases upper airway susceptibility [11,12,13].

High-flow nasal oxygen (HFNO) delivers heated, humidified gas at flows up to 60 L/min through purpose-designed nasal cannulae, enabling stable FiO_2_ delivery independent of the patient’s peak inspiratory flow [14,15]. Its physiological effects are mediated through four mechanisms: a flow-dependent positive end-expiratory pressure at the nasopharyngeal level (~0.8–1.0 cm H_2_O per 10 L/min), promoting alveolar recruitment [16,17]; nasopharyngeal washout of exhaled CO_2_-rich gas, increasing alveolar ventilation relative to minute ventilation [18]; elimination of room air entrainment that makes conventional low-flow devices unreliable [14]; and delivery of gas at body temperature and 100% relative humidity, improving mucociliary clearance and patient comfort [19].

Pooled evidence from randomised trials and meta-analyses now generally favours HFNO over conventional oxygen for the prevention of hypoxaemia during sedated endoscopy, particularly in higher risk populations; however, heterogeneity in patient selection, flow rates, and comparator oxygen strategies limits applicability across patient and procedure strata [20,21,22,23,24,25]. Despite this accumulating evidence, few studies have applied propensity score methods to correct for selection bias in real-world practice cohorts enriched for OSA risk, and no data-derived sample size estimate for a confirmatory trial in this specific population has been reported.

To address this gap, we conducted a retrospective cohort study in a high-risk population undergoing deep sedation for colonoscopy, applying PSM to control for the principal sources of clinician preference-related selection bias. The primary objective was to estimate the association between two oxygen delivery strategies (HFNO vs. Venturi mask oxygen) and procedure-related desaturation after adjustment for baseline differences. A secondary objective was to provide a data-derived sample size estimate for a prospective confirmatory randomised trial.

## 2. Materials and Methods

### 2.1. Study Design, Setting, and Ethical Approval

A single-centre retrospective cohort study was conducted at the Critical Care Department, Hospital Universitario del Tajo, Aranjuez, Spain, approved by the Ethics Committee for Clinical Trials of Hospital Universitario 12 de Octubre (Madrid, Spain) and conducted in full accordance with the Declaration of Helsinki (revised 2013) and Spanish biomedical research legislation (Ley 14/2007 and Ley Orgánica 3/2018). Given the retrospective, observational design, the Ethics Committee waived individual informed consent. This manuscript was prepared in accordance with the STROBE statement for cohort studies [26]. The authors declare no conflicts of interest.

### 2.2. Patients and Cohort Assembly

During the study period (February 2018–February 2020), 847 colonoscopies under deep sedation were performed. Of these, 200 consecutive patients met pre-specified eligibility criteria and were included; one procedure per patient was included. 

Eligibility required age ≥18 years and high risk of respiratory adverse events, defined as: (a) established diagnosis of OSA, with or without nocturnal CPAP therapy; or (b) high probability of moderate-to-severe OSA on the STOP-BANG questionnaire [27] (total score ≥ 5, or score ≥ 2 combined with one of: male sex; age > 50 years; BMI > 35 kg/m^2^; or neck circumference > 43 cm in men/>41 cm in women). These criteria represent a study-specific high-risk definition derived from the STOP-BANG instrument and do not correspond to the standard validated two-step STOP-BANG high-risk algorithm. Patients were assigned to one of seven pre-specified, mutually exclusive inclusion categories (Table 1). Exclusion criteria were: severe pulmonary disease; simultaneous gastroscopy; allergy to propofol or opioids; and anatomical impossibility of nasal cannula placement.

The oxygen delivery strategy (HFNO or Venturi mask) was selected at the discretion of the attending intensivist: The oxygen delivery strategy was determined by the scheduling allocation of the attending intensivist for each session, resulting in a near-equal distribution of procedures across strategies per clinician, as detailed in Supplementary Table S1. Both groups were enrolled concurrently throughout the study period (Supplementary Figure S1). No patient crossed over from their assigned oxygen strategy. This mechanism represents clinician preference-related selection bias rather than confounding by indication in the classical sense.

### 2.3. Oxygen Delivery Strategies and Sedation Protocol

In the HFNO group, oxygen was delivered via heated humidified high-flow nasal cannula (Optiflow^®^, Fisher & Paykel Healthcare, Irvine, CA, USA) at 50 L/min with FiO_2_ 0.50. In the control group, oxygen was administered via Venturi mask at 12 L/min (FiO_2_ 0.40). The two strategies differed simultaneously in flow rate, delivered oxygen concentration (FiO_2_ 0.50 vs. 0.40), device interface, and degree of humidification; any observed difference in desaturation rates cannot be attributed exclusively to the high-flow or positive airway pressure mechanism of HFNO. HFNO was applied before baseline SpO_2_ measurement and before sedation induction; baseline SpO_2_ was therefore measured after initiation of the assigned oxygen strategy and should be interpreted as a post-exposure variable that may reflect the early physiological effect of the intervention. Neither flow nor FiO_2_ was titrated during the procedure; adherence to the assigned strategy was 100%.

Sedation was performed using propofol (median weight-adjusted total dose: 2.5 mg/kg [IQR 2.0–3.2] in HFNO vs. 2.3 mg/kg [IQR 1.9–2.9] in control; *p* = 0.412) combined with fentanyl (1 µg/kg in both groups), titrated to deep sedation (MOAA/S score 0–1), assessed every 5 min. All procedures were performed in the left lateral decubitus position by 8 experienced intensivists. Median procedure duration was 22 min (IQR 16–32) in HFNO vs. 24 min (IQR 17–35) in control (*p* = 0.531). Capnography was not used.

### 2.4. Outcome Definition

The primary outcome was desaturation, defined as SpO_2_ < 90% at any point during the procedure, regardless of duration. SpO_2_ was monitored continuously via pulse oximetry (Masimo Radical-7, Masimo Corporation, Irvine, CA, USA), with standardised recording at 5 min intervals. Artefact was managed per unit protocol (reposition of probe and confirmation with clinical assessment before recording an event). Events were not independently adjudicated. Secondary outcomes were rescue airway manoeuvres (jaw thrust/chin lift and Guedel airway insertion), nadir SpO_2_, and procedural interruption.

Outcome data were captured in a dedicated complications registry and cross-referenced against the principal registry by patient identifier. Patients absent from the complications registry (n = 56; HFNO 27, control 29; Fisher’s exact *p* = 0.847) were assigned a primary outcome of 0 (no desaturation), consistent with the unit’s mandatory documentation protocol. This assumption rests on the protocol requirement that all desaturation events be prospectively entered into a dedicated registry at the time of occurrence; absence from the registry was therefore treated as evidence of no event rather than missing data. The balanced absence rate between groups (Fisher’s exact *p* = 0.847) makes differential under-recording unlikely, though the possibility of unrecorded events in either group cannot be excluded and represents a limitation of retrospective outcome ascertainment.

### 2.5. Propensity Score Estimation and Matching

**Figure 1:**
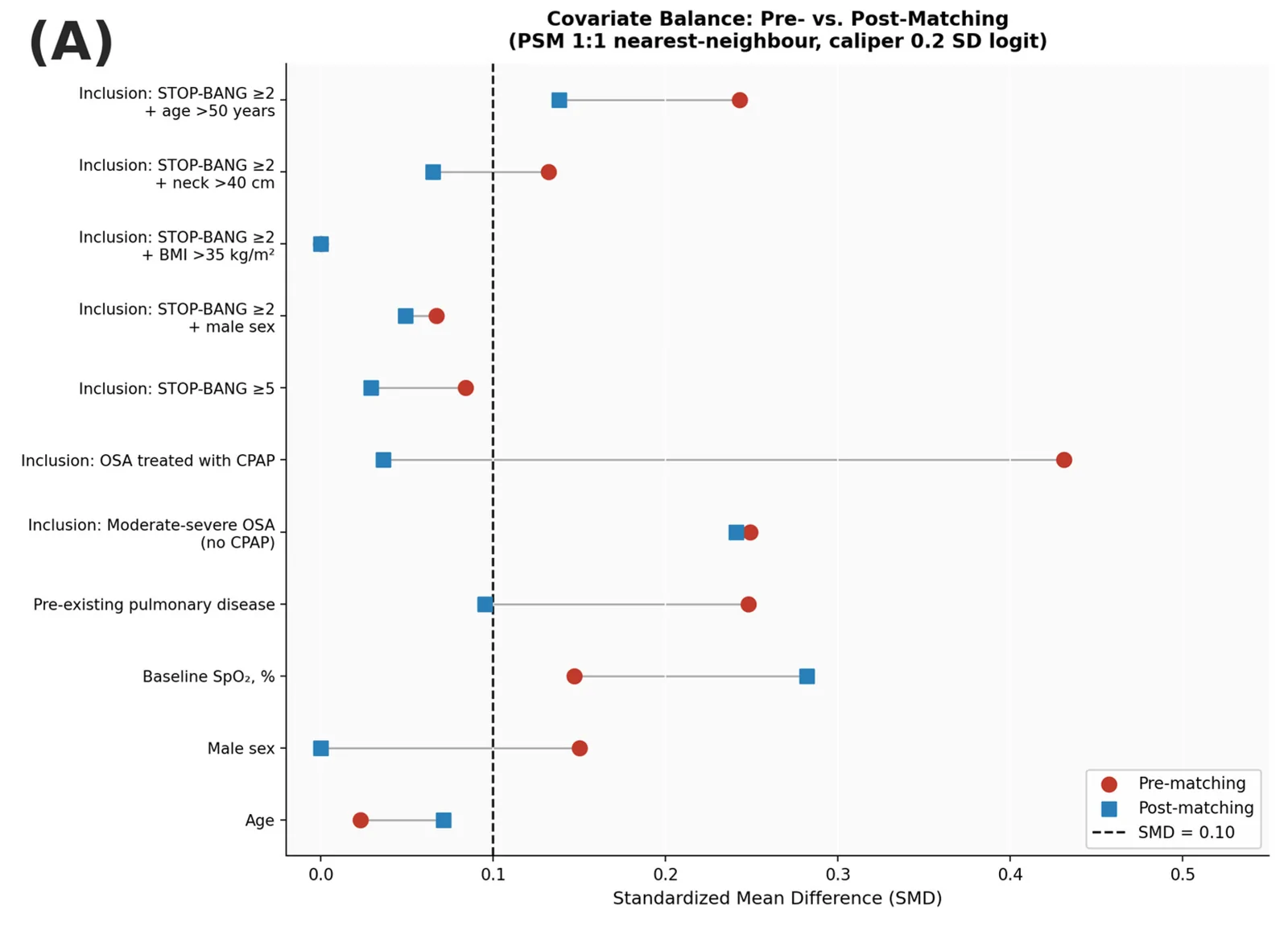

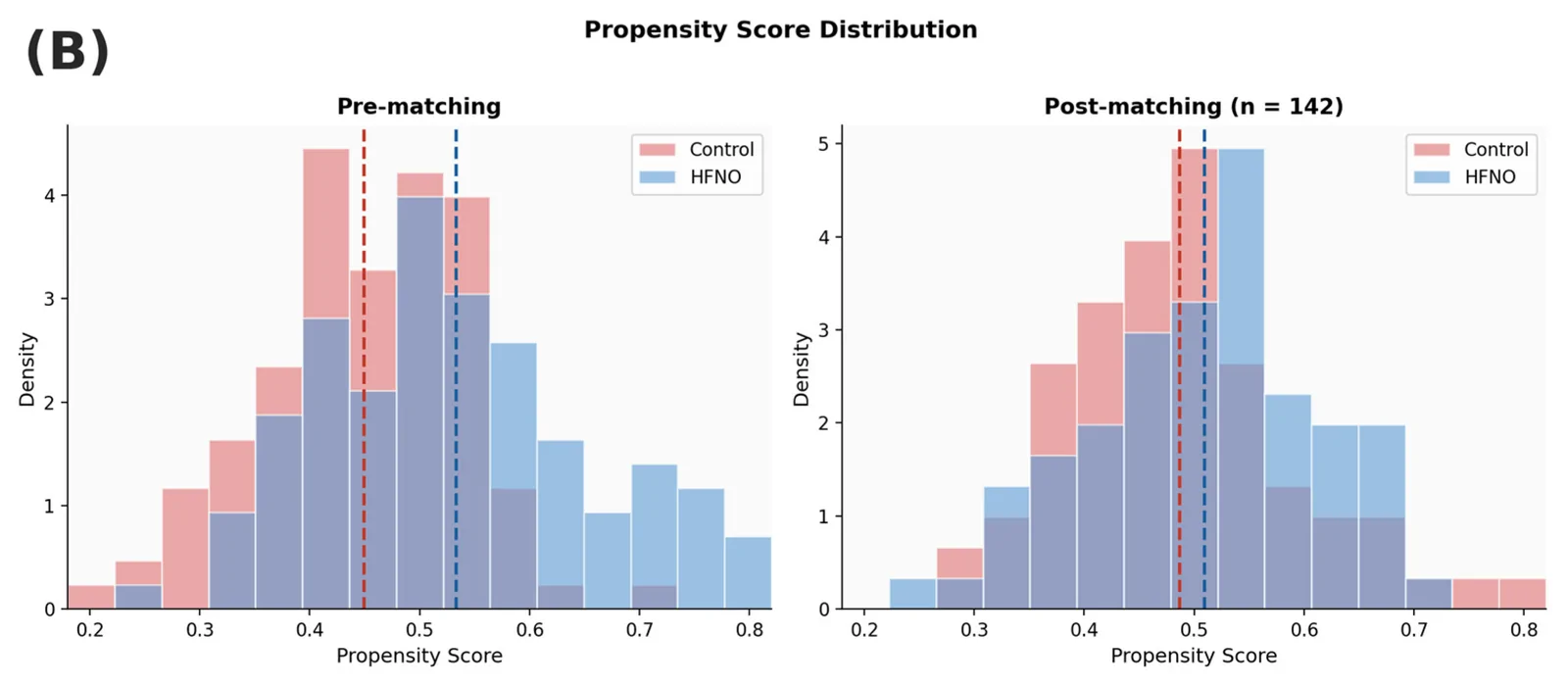
Propensity score diagnostics. (**A**) Love plot showing the standardised mean difference (SMD) for each covariate before (circles) and after (squares) matching. The dashed vertical line at SMD = 0.10 indicates the pre-specified balance threshold. (**B**) Propensity score overlap plot before matching. Solid histograms represent the pre-matching distributions; dashed-line step histograms indicate the post-matching distributions. Shaded regions indicate areas outside the common support that were excluded by the calliper.

To adjust for potential selection bias, the propensity score (PS) was estimated using multivariate logistic regression with HFNO assignment as the dependent variable and the following baseline covariates as predictors: age, sex, procedure indication (seven-level categorical variable, dummy-coded), baseline SpO_2_ (included as a covariate despite being a post-exposure variable; its retention in the PS model is acknowledged as a limitation), and pre-existing pulmonary disease. The target estimand was the average treatment effect in the treated (ATT). The AUC of the PS model is reported descriptively; PS model quality was assessed by post-matching covariate balance (Figure 1A, Table 1). Common support was assessed via overlap of PS distributions (Figure 1B).

Matching was performed using a 1:1 nearest-neighbour algorithm without replacement, with a calliper width of 0.2 standard deviations of the logit of the propensity score [28]. Treated (HFNO) patients were randomly ordered before matching to avoid systematic order bias. Covariate balance was assessed using the SMD (primary metric); SMD <0.10 was considered indicative of adequate balance [29].

### 2.6. Statistical Analysis

Continuous variables are presented as mean (SD); categorical variables as frequency and percentage. In the matched cohort, the primary outcome was analysed using the McNemar test, with the matched-pair OR and 95% CI estimated from the discordant pairs. The absolute risk difference (ARD) and its 95% CI were estimated using the Newcombe method. As a pre-specified sensitivity analysis, conditional logistic regression (CLR) was applied to the 71 matched pairs, conditioning on pair membership. A worst-case sensitivity analysis assigned desaturation to all 56 patients absent from the complications registry. A post hoc power analysis was performed to inform prospective trial planning. All analyses were performed using Python 3.11; a two-tailed *p*-value < 0.05 was considered statistically significant.

## 3. Results

### 3.1. Patient Characteristics and Pre-Matching Comparability

During the study period, 847 colonoscopies under deep sedation were screened; 200 patients met eligibility criteria and were included. The mean age was 63.4 (SD 11.2) years; 32.5% were male. Most patients were ASA physical status II (38.5%) or III (55.0%) (Table 1). The three most common indication categories were STOP-BANG ≥ 5 (35.0%), OSA treated with CPAP (24.0%), and STOP-BANG ≥ 2 + age > 50 years (22.0%).

Before matching, the two groups differed meaningfully in indication categories (OSA treated with CPAP: SMD 0.43; STOP-BANG ≥ 2 + age > 50 years: SMD 0.24; moderate-severe OSA: SMD 0.25) and in the prevalence of pre-existing pulmonary disease (HFNO 8% vs. control 16%; SMD 0.25), consistent with clinician preference-related selection bias (Table 1; Figure 1A).

### 3.2. Propensity Score Model and Matching

The logistic regression model yielded an AUC of 0.682, reported descriptively; PS overlap is shown in Figure 1B, demonstrating adequate common support. The 1:1 nearest-neighbour algorithm with a calliper of 0.1223 logit units yielded 71 matched pairs (142 patients); 29 patients from each group were excluded due to absence of a suitable match within the calliper.

### 3.3. Covariate Balance After Matching

After matching, 10 of 17 covariates achieved SMD < 0.10 (Table 1; Figure 1A). The most pronounced residual imbalance was in baseline SpO_2_ (SMD 0.28), reflecting the very low variance of this variable across the sample (range 93–100%; absolute mean difference ~0.5 percentage points, clinically inconsequential). Body weight (SMD 0.18), ASA class categories, moderate-severe OSA (SMD 0.24), and STOP-BANG ≥ 2 + age > 50 years (SMD 0.14) also remained above the 0.10 threshold, representing acknowledged sources of potential residual confounding.

### 3.4. Primary Outcome: Desaturation

In the unmatched cohort, desaturation occurred in 11 of 100 HFNO patients (11.0%) and 19 of 100 controls (19.0%) (crude OR 0.53; *p* = 0.166, chi-square).

**Figure 2:**
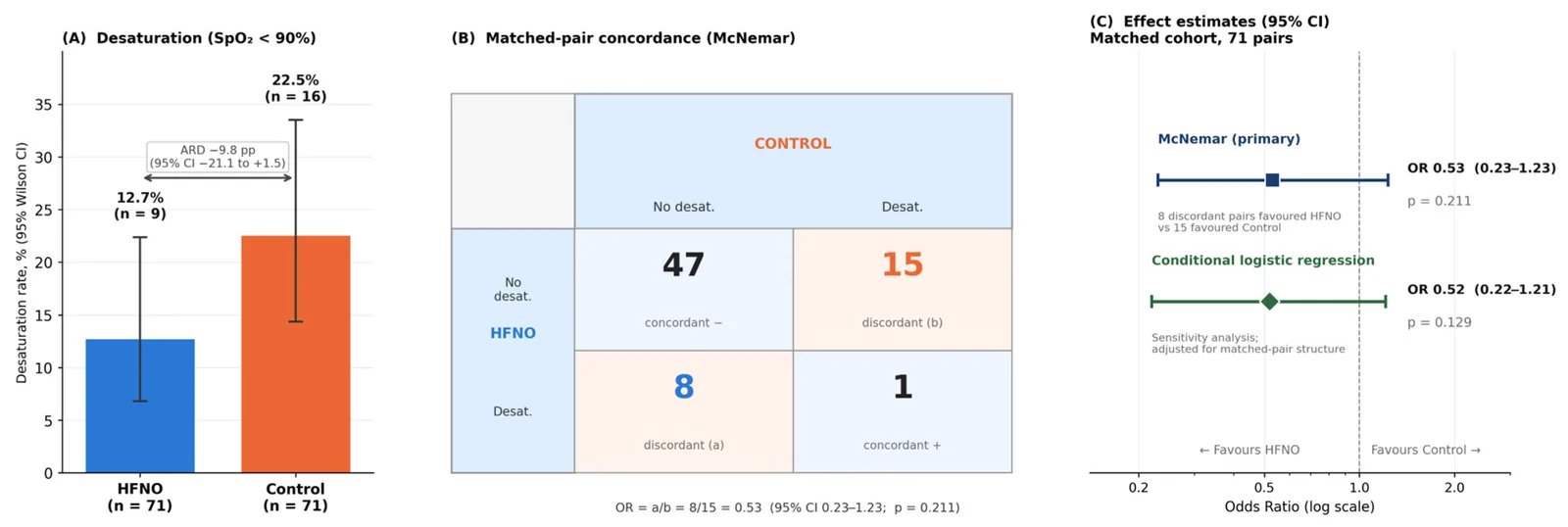
Primary outcome and sensitivity analysis in the propensity-matched cohort (71 pairs). (**A**) Desaturation rate (SpO_2_ < 90% at any time) in the HFNO and control groups. ARD = absolute risk difference. (**B**) Concordance (2 × 2) table of matched pairs. Blue-highlighted cells indicate discordant pairs (AB: HFNO desaturates, control does not; BA: control desaturates, HFNO does not), which determine the matched-pair OR (8/15 = 0.53). (**C**) Effect estimates with 95% confidence intervals for the McNemar primary analysis (square) and the conditional logistic regression sensitivity analysis (diamond), on a log scale. Neither estimate reached statistical significance.

In the matched cohort (71 pairs), desaturation was recorded in 9 of 71 HFNO patients (12.7%) and 16 of 71 controls (22.5%). The matched-pair OR was 0.53 (95% CI 0.23–1.23; *p* = 0.211) and the absolute risk difference was 9.8 percentage points (95% CI −1.5 to +21.1). The estimate was imprecise and the confidence interval included no association (Figure 2A–C).

Worst-case sensitivity analysis (all 56 absent patients assigned desaturation = 1): matched-pair OR 0.61 (95% CI 0.28–1.31; *p* = 0.289), consistent in direction and similarly inconclusive.

### 3.5. Sensitivity Analysis: Conditional Logistic Regression

CLR applied to the 71 matched pairs, conditioning on pair membership, yielded an adjusted OR of 0.52 (95% CI 0.22–1.21; *p* = 0.129), confirming the direction of the McNemar estimate (Figure 2C). Neither analysis reached statistical significance.

### 3.6. Secondary Outcomes

Secondary outcome analyses were performed in the full unmatched cohort (n = 200); these analyses are exploratory and should be interpreted with caution.

Rescue airway manoeuvres: jaw-thrust or chin-lift manoeuvres were required in 17.2% of control patients versus 5.0% of HFNO patients (*p* = 0.006, Fisher’s exact; ARD −12.2 pp, 95% CI −21.0 to −3.4). Guedel airway insertion was needed in 11.1% versus 4.4% (*p* = 0.057; ARD −6.7 pp, 95% CI −14.5 to +1.1); this difference did not reach statistical significance. These manoeuvres were assessed in an unblinded setting, introducing the possibility of differential intervention thresholds. No patient required bag-mask ventilation, orotracheal intubation, or laryngeal mask airway placement.

Nadir SpO_2_: median nadir SpO_2_ was 98% (IQR 96–99%) in HFNO patients versus 96% (IQR 93–99%) in controls in the full cohort (*p* = 0.031); in the matched cohort: 98% (IQR 96–99%) vs. 95% (IQR 92–99%), *p* = 0.058.

Procedural interruption: endoscopy was interrupted in one HFNO patient (1.0%) and five control patients (5.0%) in the full cohort (*p* = 0.210); in the matched cohort: 1/71 (1.4%) vs. 3/71 (4.2%), *p* = 0.617.

### 3.7. Sample Size Estimation for Future Trial Planning

Post hoc power analysis indicated that the current 71 matched pairs provided 30.8% statistical power. Achieving 80% power would require approximately 265 matched pairs; 90% power would require 355 matched pairs (Table 2). These estimates are provisional and derived from an underpowered current sample.

## 4. Discussion

### 4.1. Principal Findings

The primary finding of this study is that the direction of the observed association favoured HFNO over Venturi mask oxygen in terms of desaturation during deep sedation for colonoscopy in patients at increased respiratory risk, but the estimate was imprecise (OR 0.53, 95% CI 0.23–1.23; ARD 9.8 pp, 95% CI −1.5 to +21.1), the confidence interval included no association, and neither the primary nor the sensitivity analysis reached statistical significance. We therefore interpret this study as hypothesis-generating rather than confirmatory; it cannot establish the efficacy of HFNO or demonstrate causality. We consider an absolute reduction of ≥10 percentage points in desaturation rate clinically important based on the prior literature; the observed ARD is consistent with this threshold but the CI encompasses both clinically important benefit and no clinically relevant effect.

### 4.2. Contextualisation Within the Literature

The desaturation rate observed in the control group (19% before matching, 22.5% after) is consistent with rates in similarly selected high-risk populations. Thiruvenkatarajan et al. [30], in a multicentre RCT of patients with ASA ≥ III, BMI >30 kg/m^2^, or suspected OSA (n = 132), reported 9.1% vs. 7.7% (*p* = 0.77). Riccio et al. [25], in morbidly obese patients (mean BMI ~48 kg/m^2^), found 45.2% in controls. Our intermediate rate reflects the moderate-to-high risk profile of our population and the Venturi mask comparator (FiO_2_ 0.40), which provides higher oxygenation than the low-flow nasal cannulae used in some other trials [23,30,31,32,33], and may have attenuated any detectable difference.

The matched-pair OR of 0.53 is numerically similar to the high-risk subgroup estimates reported by Nay et al. [34] and falls within the range of estimates from recent meta-analyses [21,22]. The 2024 network meta-analysis by the most current high-quality evidence from recent systematic reviews and randomised trials supports HFNO in higher risk populations [35,36,37,38,39]. Our observational data are consistent with this direction but do not add confirmatory evidence.

### 4.3. Selection Bias, PSM, and Residual Confounding

The mechanism of oxygen strategy allocation—individual clinician preference—generated systematic baseline differences before matching (OSA + CPAP: SMD 0.43; pulmonary disease: SMD 0.25), consistent with clinicians who routinely used HFNO having a different patient profile from those who used Venturi masks. PSM reduced most imbalances (10/17 covariates with SMD < 0.10), but residual imbalance remained for baseline SpO_2_ (SMD 0.28), body weight (SMD 0.18), and several ASA and indication categories. These imbalances, together with unmeasured confounders—including STOP-BANG score, Mallampati class, and neck circumference—that could not be incorporated into the PS model, limit causal inference.

The exposure comparison is further limited by the simultaneous difference in FiO_2_ (0.50 vs. 0.40) between strategies. The higher FiO_2_ in the HFNO group may independently contribute to any observed reduction in desaturation, independent of flow rate or positive airway pressure. The results should be interpreted as reflecting the total effect of the HFNO strategy versus the Venturi mask strategy, not the isolated effect of high flow.

### 4.4. Statistical Power and Implications for Trial Design

The current sample provided only 30.8% statistical power. Underpowering arose from two compounding factors: only 71 of 200 patients yielded matched pairs, and only 23 of 71 pairs were discordant—the only pairs contributing information to the McNemar test. The 95% CI (0.23–1.23) reflects this uncertainty. A future prospective parallel group randomised trial would require approximately 237 patients per group (474 total), calculated using a two-proportion z-test with assumed desaturation rates of 12.7% (HFNO) and 22.5% (control), two-tailed α = 0.05, and 80% power. These parameters should be treated as provisional estimates derived from an underpowered current sample. These parameters should be treated as provisional estimates based on an underpowered current sample.

### 4.5. High-Risk Population, OSA, and the Two-Strategy Comparison

The selection of patients based on OSA diagnosis or high STOP-BANG score is physiologically justified: sedative agents blunt microarousal reflexes that protect against pharyngeal collapse during sleep, and muscle relaxation further increases upper airway susceptibility [11]. HFNO may confer particular physiological benefit in this population through its PEEP-like nasopharyngeal effect (~3–5 cm H_2_O at 50 L/min with closed mouth) and dead space washout [16]; however, the present study, using the Venturi mask oxygen at FiO_2_ 0.40, cannot isolate this mechanism from the FiO_2_ effect. In the data underlying this cohort, patients who desaturated were disproportionately drawn from the highest risk categories (OSA + CPAP or STOP-BANG ≥ 5: 72.7% of desaturating HFNO patients; 57.9% of desaturating controls), consistent with Riccio et al. [25] and with current guideline recommendations for HFNO in high-risk patients.

### 4.6. Discussion of Secondary Outcomes

The statistically significant reduction in jaw-thrust manoeuvres in the full cohort (17.2% vs. 5.0%; *p* = 0.006; ARD −12.2 pp, 95% CI −21.0 to −3.4) adds a potentially clinically important dimension, but must be interpreted cautiously given the unmatched analysis, unblinded assessment, and the possibility of differential intervention thresholds between the two groups of intensivists. The reduction in nadir SpO_2_ (*p* = 0.031 in the full cohort; *p* = 0.058 in the matched cohort) and the directionally consistent results for procedural interruption are supportive but similarly inconclusive. These findings are consistent with Nay et al. [34] and with the meta-analytic reduction in rescue airway interventions reported by Hung et al. [21].

### 4.7. Limitations

This study has several limitations. First, its retrospective single-centre design limits the generalisability of our findings to other settings and patient populations.

Second, allocation was not randomised but reflected clinician preference, so residual confounding is likely despite propensity score matching. This concern is reinforced by two further observations: seven of the 17 covariates retained a standardised mean difference of 0.10 or greater after matching, and the propensity score model included only five covariate categories. It did not account for the STOP-BANG score, Mallampati class, neck circumference or weight-adjusted sedative dose, all of which may influence both treatment choice and outcome.

Third, the two strategies also differed in FiO_2_, which prevents us from attributing the observed effect to a specific mechanism. Similarly, capnography was not available, so ventilation and hypercapnia could not be assessed.

Fourth, outcome ascertainment has its own weaknesses. Completeness of the primary outcome depended on the quality of clinical documentation. Secondary outcomes were analysed in the unmatched cohort only, and rescue manoeuvres were assessed without blinding, which may have introduced detection bias.

Finally, the sample was underpowered, and the post hoc power estimates derived from it carry substantial uncertainty and should be interpreted with caution. Sixth, baseline SpO_2_ was measured after initiation of the assigned oxygen strategy and therefore constitutes a post-exposure variable. Its inclusion in the propensity score model may have introduced bias; excluding SpO_2_ from the PS model is a sensitivity analysis that future work should report.

### 4.8. Clinical and Research Implications

This study provides three contributions. First, it empirically demonstrates and quantifies clinician preference selection bias in real-world HFNO allocation within a single unit, showing that PSM substantially reduces—but does not eliminate—this bias. Second, it provides provisional trial design parameters (474 patients, 237 per group, parallel group; OR 0.53) for a prospective confirmatory study in an OSA/STOP-BANG-enriched population. Third, it shows that even with a more rigorous Venturi mask comparator (FiO_2_ 0.40), the observed direction of effect is consistent with the existing randomised evidence, including the existing randomised trial evidence and current guideline recommendations.

## 5. Conclusions

In this retrospective propensity score-matched cohort of patients at increased respiratory risk undergoing deep sedation for colonoscopy, the observed desaturation rate was lower with HFNO than with the Venturi mask oxygen (OR 0.53, 95% CI 0.23–1.23; ARD 9.8 pp, 95% CI −1.5 to +21.1 pp). However, the estimate was imprecise, the confidence interval included no association, and residual confounding, incomplete covariate balance, and the simultaneous FiO_2_ difference between strategies preclude definitive conclusions. These findings should be considered hypothesis-generating. A prospectively designed, adequately powered parallel group randomised trial—with an estimated sample size of approximately 474 patients (237 per group)—is required to determine whether HFNO reduces desaturation in this population.

## Figures and Tables

**Table 1:** Baseline characteristics and primary outcome of the study population before and after propensity score matching, stratified by oxygen delivery strategy. Continuous variables: mean (SD). Categorical variables: n (%). SMD: standardised mean difference. SMD is the primary balance metric; *p*-values are provided for completeness only. Red SMD values (SMD ≥ 0.10): residual imbalance; green SMD values (SMD < 0.10): adequate balance. HFNO: high-flow nasal oxygen; OSA: obstructive sleep apnoea; CPAP: continuous positive airway pressure; BMI: body mass index.

Variable	HFNO (n = 100)	Control (n = 100)	SMD	*p*	HFNO (n = 71)	Control (n = 71)	SMD	*p*
**Continuous Variables**								
Age, years	63.6 (11.5)	63.3 (10.9)	0.023	0.752	63.6 (11.4)	62.8 (11.1)	0.071	0.630
Body weight, kg	83.2 (13.6)	83.6 (15.2)	0.026	0.774	82.0 (13.2)	84.7 (16.1)	0.183	0.281
BMI, kg/m^2^	30.5 (4.6)	30.3 (4.9)	0.043	0.643	30.5 (4.8)	30.7 (4.9)	0.044	0.893
Baseline SpO_2_, %	99.1 (1.5)	99.3 (1.2)	0.147	0.526	98.9 (1.7)	99.4 (1.1)	0.282	0.191
**Categorical variables**	—	—	—	—	—	—	—	—
Male sex, n (%)	29 (29.0%)	36 (36.0%)	0.150	0.365	25 (35.2%)	25 (35.2%)	0.000	1.000
Pre-existing pulmonary disease, n (%)	8 (8.0%)	16 (16.0%)	0.248	0.128	8 (11.3%)	6 (8.5%)	0.095	0.778
**ASA Physical Status, n (%)**								
ASA I	0(0.0%)	2 (2.0%)	0.202	0.497	0(0.0%)	2 (2.8%)	0.241	0.496
ASA II	34 (34.0%)	43 (43.0%)	0.186	0.245	26 (36.6%)	32 (45.1%)	0.173	0.393
ASA III	60 (60.0%)	50 (50.0%)	0.202	0.201	41 (57.7%)	34 (47.9%)	0.198	0.313
ASA IV	6 (6.0%)	5 (5.0%)	0.044	1.000	4 (5.6%)	3 (4.2%)	0.065	1.000
**Inclusion Criterion, n (%)**								
Moderate-severe OSA (no CPAP)	0(0.0%)	3 (3.0%)	0.249	0.246	0(0.0%)	2 (2.8%)	0.241	0.496
OSA treated with CPAP	33 (33.0%)	15 (15.0%)	0.431	0.005	13 (18.3%)	14 (19.7%)	0.036	1.000
STOP-BANG ≥5	33 (33.0%)	37 (37.0%)	0.084	0.657	29 (40.8%)	30 (42.3%)	0.029	1.000
STOP-BANG ≥2 + male sex	11 (11.0%)	9 (9.0%)	0.067	0.814	6 (8.5%)	7 (9.9%)	0.049	1.000
STOP-BANG ≥2 + BMI >35 kg/m^2^	2 (2.0%)	2 (2.0%)	0.000	1.000	2 (2.8%)	2 (2.8%)	0.000	1.000
STOP-BANG ≥2 + neck >40 cm	4 (4.0%)	7 (7.0%)	0.132	0.537	4 (5.6%)	3 (4.2%)	0.065	1.000
STOP-BANG ≥2 + age >50 years	17 (17.0%)	27 (27.0%)	0.243	0.124	17 (23.9%)	13 (18.3%)	0.138	0.537
**Primary Outcome**								
Desaturation (SpO_2_ < 90%), n (%)	11 (11.0%)	19 (19.0%)	0.225	0.166	9 (12.7%)	16 (22.5%)	0.261	0.186

**Table 2:** Sample size estimation for prospective trial planning. Assumptions: discordant-pair proportion = 32.4% (observed); OR = 0.53 (observed); α = 0.05 (two-tailed). Estimates are provisional.

Matched Pairs (N)	Estimated Discordant Pairs	Statistical Power	Interpretation
71	23	30.8%	Current study
100	32	41.0%	
150	49	56.4%	
200	65	68.8%	
265	86	80.5%	Target minimum (80% power)
355	115	90.4%	90% power
500	162	97.2%	

## Data Availability

The data supporting the findings of this study are not publicly available due to privacy and ethical restrictions but are available from the corresponding author upon request to the Editor-in-Chief.

## Supplementary Materials

The following supporting information can be downloaded at: https://www.mdpi.com/article/10.3390/jcm15218254/s1, Table S1: Number of colonoscopy procedures performed per intensivist during the study period (February 2018–February 2020). Figure S1: STROBE-compliant participant flow diagram.
