# Supplementary material for: High-Flow Nasal Oxygen and Desaturation During Deep Sedation for Colonoscopy in Patients at Increased Respiratory Risk: A Propensity Score-Matched Cohort Study
Source: J Clin Med. 2026 Oct 23;15(21):8254. doi: 10.3390/jcm15218254 (PMC13764395; doi:10.3390/jcm15218254)
Supplement: Supplementary file 1 [file jcm-15-08254-s001.zip › jcm-4536388-supplementary.pdf]

Supplementary Table S1. Number of colonoscopy procedures performed per intensivist during the study period (February 2018–February 2020).

Intensivist	HFNO procedures (n)	Control procedures (n)	Total procedures (n)	% of total cohort
1	13	12	**25**	12.5%
2	13	12	**25**	12.5%
3	13	13	**26**	13.0%
4	12	13	**25**	12.5%
5	13	12	**25**	12.5%
6	12	13	**25**	12.5%
7	12	13	**25**	12.5%
8	12	12	**24**	12.0%
**Total**	**100**	**100**	**200**	**100.0%**

HFNO, high-flow nasal oxygen.

Intensivists are anonymised using sequential identifiers (1 to 8). Identifiers are not linked to any other variable in the dataset.

All 8 intensivists had equivalent experience with both oxygen-delivery strategies (HFNO via Optiflow® device and Venturi-mask oxygen) before the study period, as recorded in the institutional training log. Procedure allocation reflected scheduling availability, not individual preference or expertise.

The small number of procedures per clinician (range 24–26; median 25) precluded formal statistical adjustment for clinician clustering.

Supplementary Figure S1. STROBE-compliant participant flow diagram

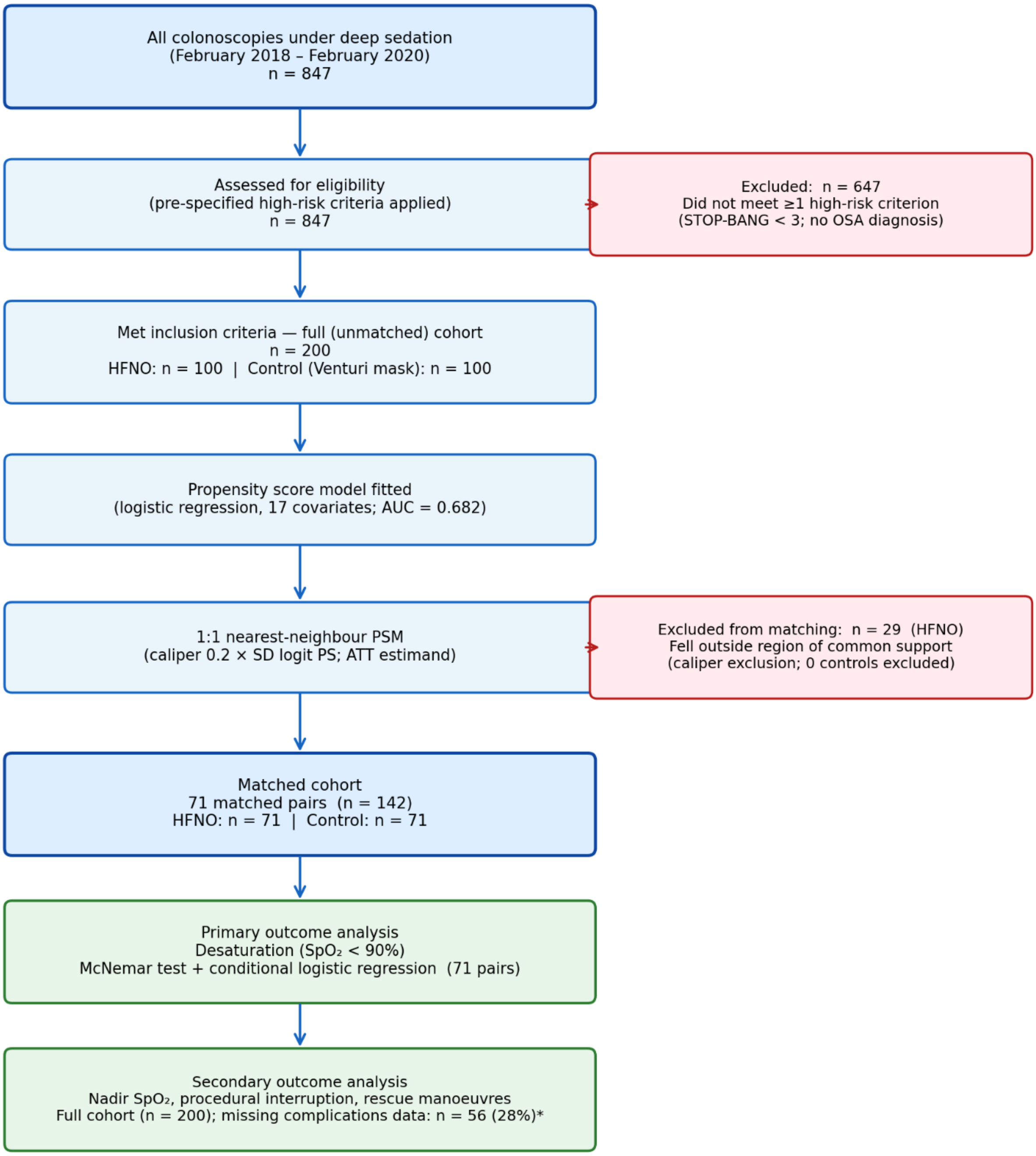

** Missing complications registry entries were cross-referenced against the principal registry ("Procedimiento completado = 1"); balanced between groups (27 HFNO vs. 29 control; p = 0.847). Sensitivity analysis (all 56 absent = desaturation): matched-pair OR 0.61 (95% CI 0.28–1.31; p = 0.289).*
